# Supplementary material for: Rapid identification of bacterial isolates using microfluidic adaptive channels and multiplexed fluorescence microscopy
Source: Lab Chip. 2024 Sep 6;24(20):4843–58. doi: 10.1039/d4lc00325j (PMC11409657; doi:10.1039/d4lc00325j)
Supplement: LC-024-D4LC00325J-s001 [file LC-024-D4LC00325J-s001.pdf]

Supplementary Information (SI) for Lab on a Chip.
This journal is © The Royal Society of Chemistry 2024

Supplementary Information: Rapid Identification of Bacterial Isolates Using Microfluidic Adaptive Channels and Multiplexed Fluorescence Microscopy

Stelios Chatzimichail[a,b*], Piers Turner[a,b], Conor Feehily[c], Alison Farrar[a,b], Derrick Crook[c,d], Monique Andersson[c,d], Sarah Oakley[c,d], Lucinda Barrett[c,d], Hafez El Sayyed[a,b], Jingwen Kyropoulos[a], Christoffer Nellåker[e], Nicole Stoesser[c,d], Achillefs N. Kapanidis[a,b*]

a. Department of Physics, University of Oxford, Parks Road, Oxford, OX1 3PJ, United Kingdom
b. Kavli Institute for Nanoscience Discovery, University of Oxford, South Parks Road, Oxford OX1 3QU, United Kingdom
c. Nuffield Department of Medicine, University of Oxford, John Radcliffe Hospital, Oxford, OX3 9DU, United Kingdom
d. Department of Microbiology and Infectious Diseases, Oxford University Hospitals NHS Foundation Trust, Oxford, OX3 9DU, United Kingdom
e. Nuffield Department of Women's & Reproductive Health, University of Oxford, Big Data Institute, Oxford, OX3 7LF, United Kingdom

Table of contents:

- **Figure S1:** The layout of the ACBC microfluidic device featuring all dimensions used.
- **Figure S2:** Channel profiles resulting from 3D printed moulds.
- **Figure S3**: Fluorescence measurements of adaptive channel height as a function of applied pressure.
- **Figure S4**: Visualization of the capture region.
- **Figure S5**: Fluidic setup used for ACBC chip capture of bacteria.
- **Figure S6**: Permeabilization of a pathogenic *E. coli* isolate in TEG buffer.
- **Figure S7**: Benchtop fabrication procedure of the ACBC chip.
- **Table S1**: Table containing the encoded FISH probes used for species identification.
- **Table S2**: Table containing the imager FISH probes used for species identification.
- **Table S3**: Table containing classification rates breakdown by bacterial strain tested.
- **Table S4**: Tabulated assay characteristics of the Multiplexed 16S-rRNA FISH assay.
- **Table S5**: Tabulated performances of rapid bacterial identification platforms.
- **Table S6:** Components and cost-breakdown table for the fluidic setup used.

Layout of the ACBC microfluidic device

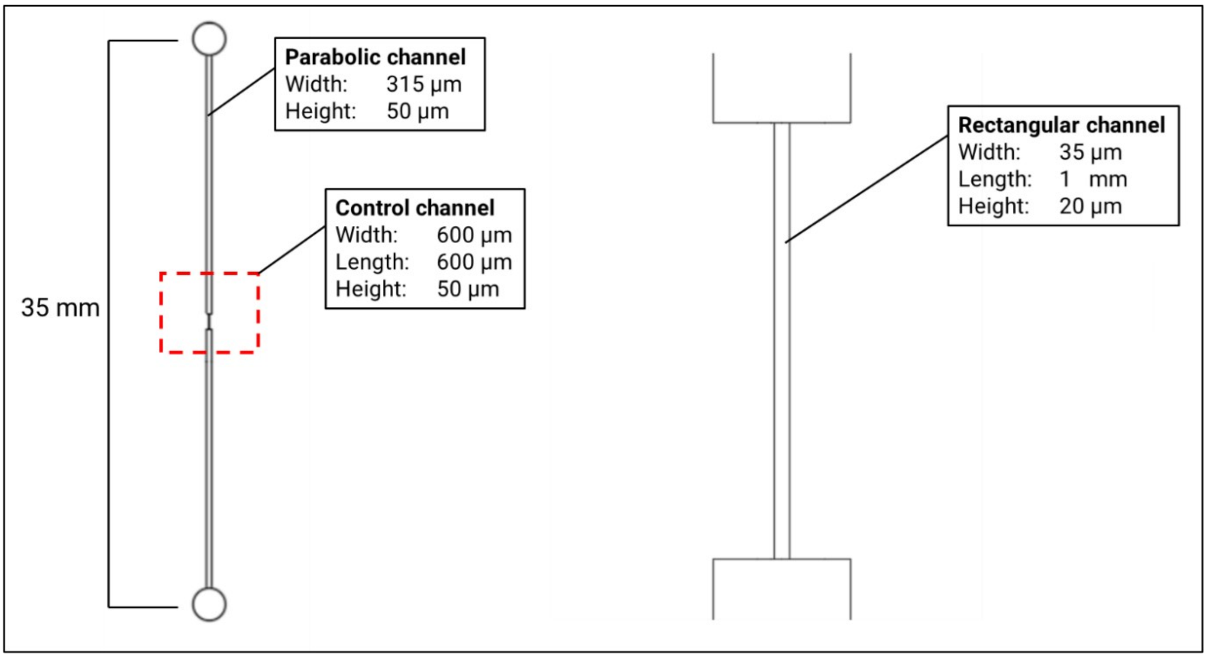

Figure S1: The layout of the ACBC microfluidic device featuring all dimensions used.

Channel profiles resulting from 3D printed moulds

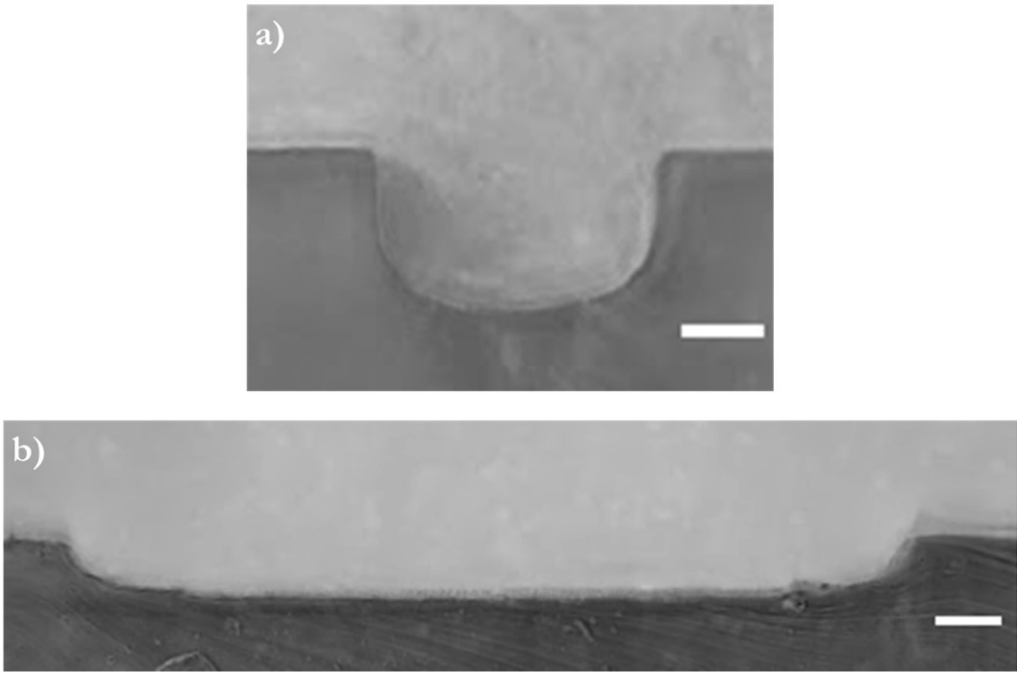

Figure S2: Channel cross-section images of the ACBC device. **a)** Cross-section of the rectangular channel (scale bar is 10 µm) and **b)** parabolic channel (scale bar is 30 µm). The angle of the PDMS walls relative to where the substrate is to be placed was determined to be 90° for the narrow channel and 60° for the parabolic channel.

Fluorescence measurements of adaptive channel height as a function of applied pressure:

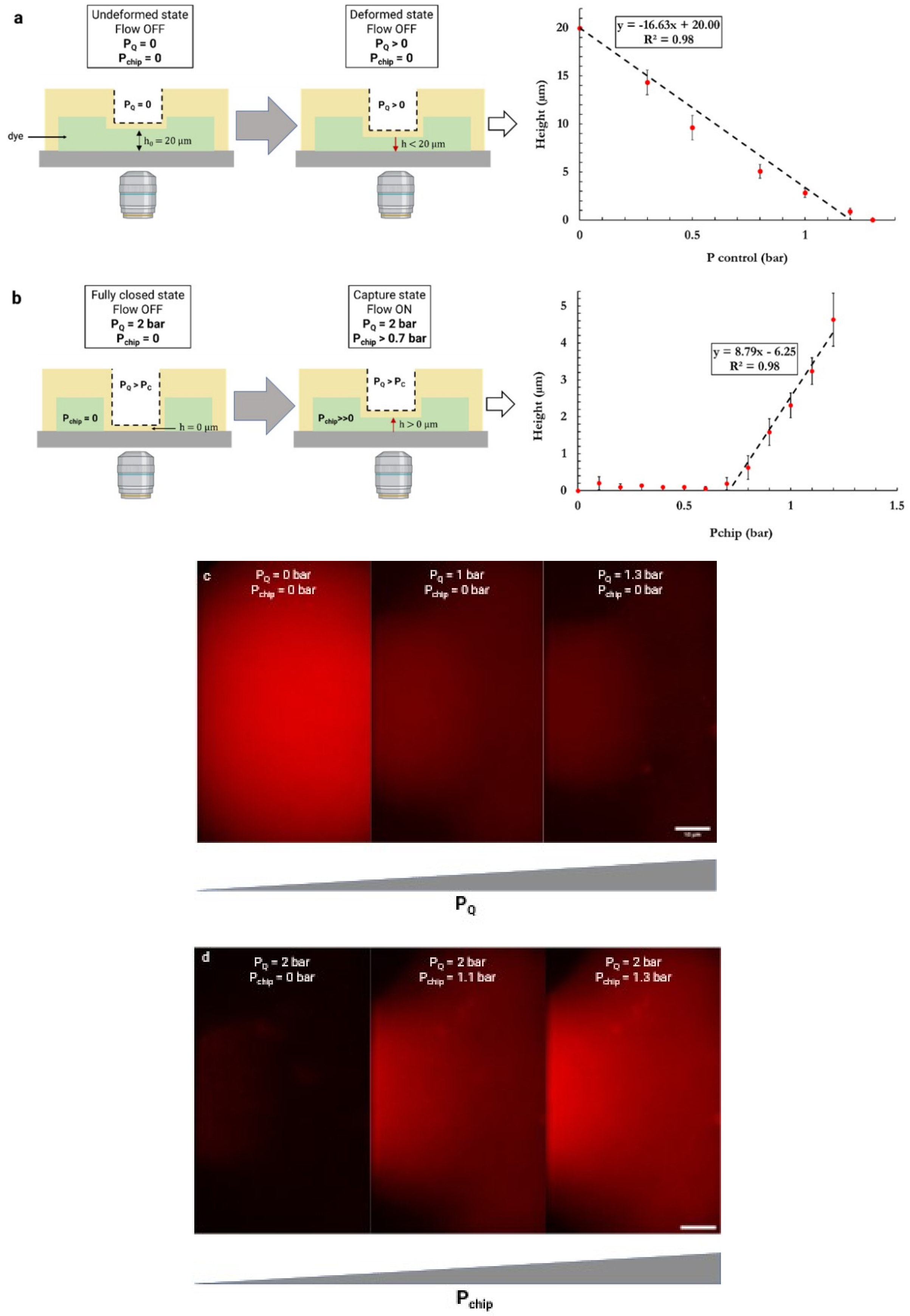

Figure S3: Estimation of ACBC channel height using epifluorescence measurements. **a)** 'Close-down' experiments schematic and fluorescence intensity based estimated height of the adaptive channel. The pressure in the control layer is varied with no flow-induced pressure and fluorescence of the dye is acquired. The fluorescence estimates suggest that each bar of pressure changes the channel height by 16.6 µm. **b)** 'Open-up' experiments schematic where the pressure in the control layer is kept constant at 2 bar and flow-induced pressure is varied. It is observed that the height remains collapsed until a pressure of 0.7 bar inside the chip is generated, after which a linear increase in channel height with flow-induced pressure is observed, with a regression coefficient of 8.8 µm/bar. **c)** and **d)** Epifluorescence images for the 'close-down' and 'open-up' experiments at three different conditions. Scale bar is 10 µm.

Visualization of the capture region

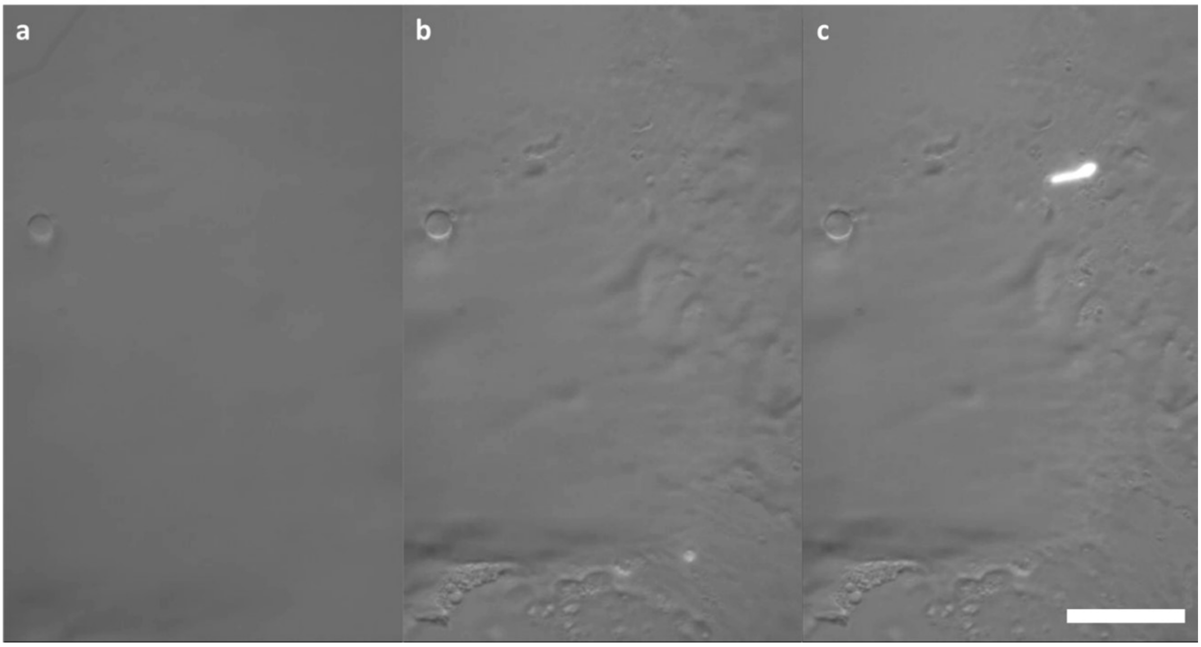

Figure S4: Visualization of capture region boundary in HILO mode using 473 nm excitation wavelength. a) The channel is in its original design dimensions. b) The channel is actuated and a capture region is formed. The geometry of the PDMS membrane can be observed. c) An incoming, auto-fluorescent, *E. coli* cell is shown captured in the capture region. The scale bar corresponds to 15 µm.

Permeabilization assessment: Pathogenic *E. coli* isolate in TEG buffer

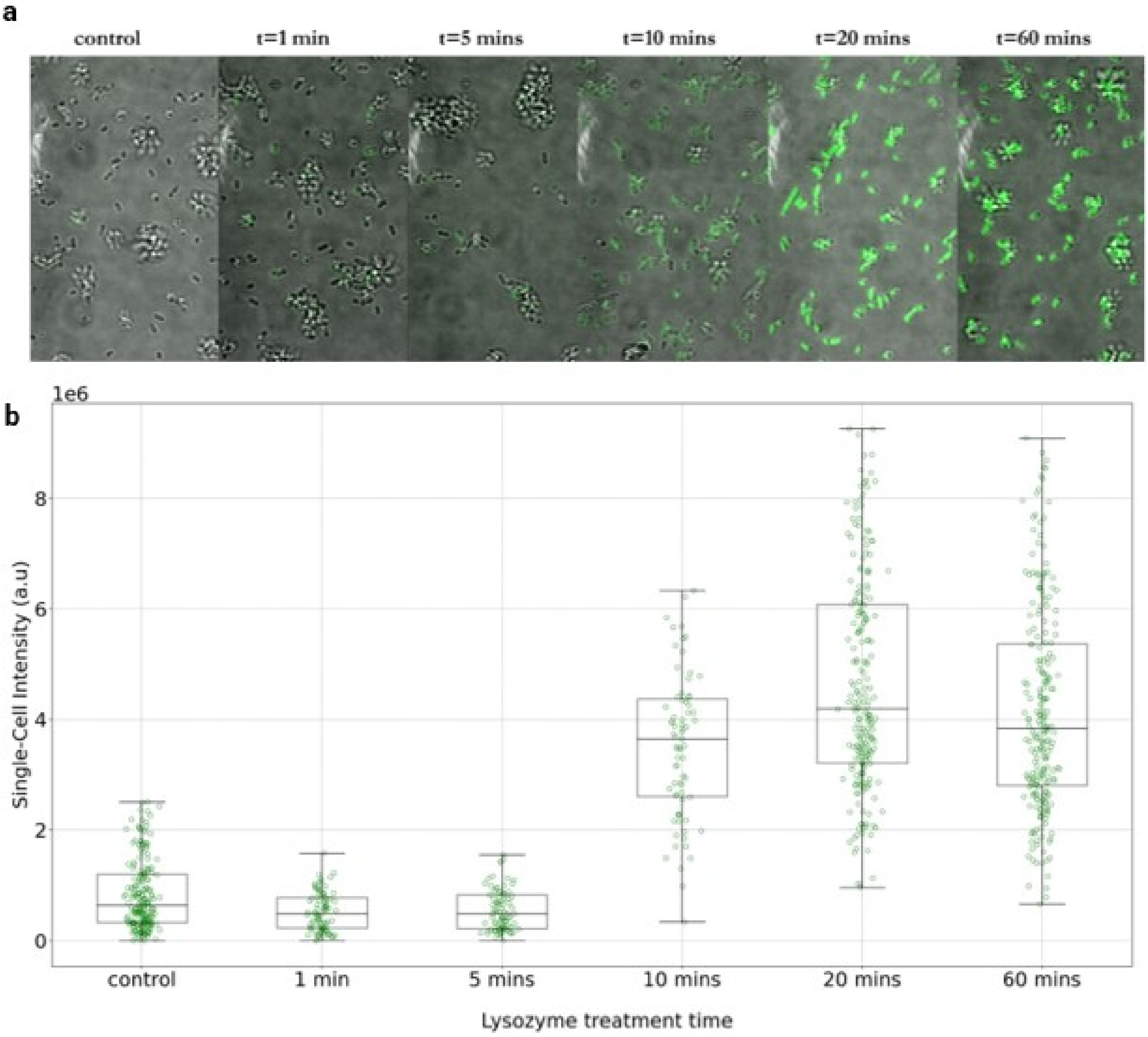

Figure S5: (a) Representative images and **(b)** EUB338-Cy3 single-cell intensity boxplots showing the effect of lysozyme incubation time (20 mg/mL, TEG buffer) for a fixed + ethanol treated pathogenic *E. coli* isolate strain. The accessibility of the EUB338-Cy3 probe was used as a proxy to evaluate cell permeabilization as a function of lysozyme treatment time. It appeared that a 20-minute treatment time was sufficient to achieve full permeabilization of this strain. Furthermore, incubation over a longer period (60 minutes) did not appear to have a deleterious effect on 16S rRNA content.

Fluidic setup used for ACBC chip capture of bacteria.

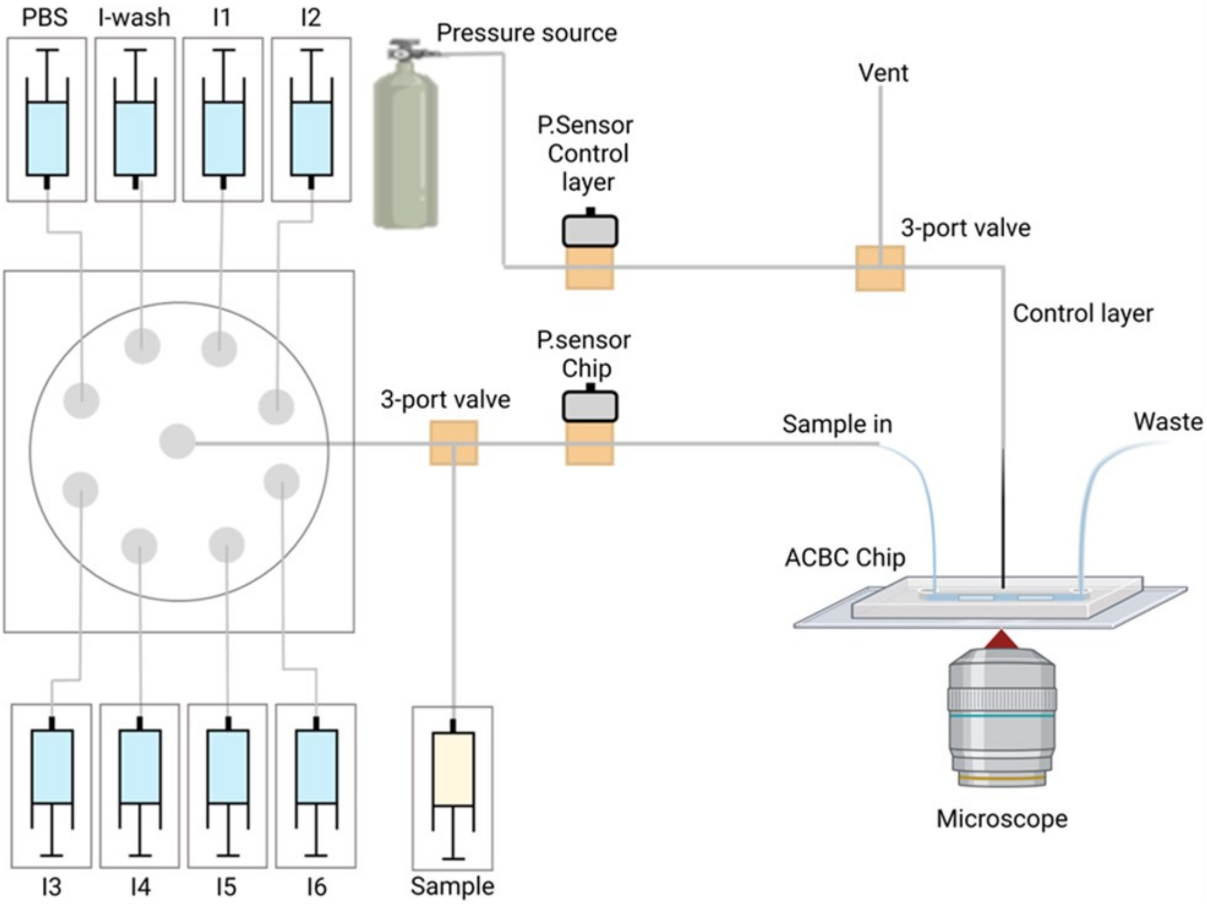

Figure S6: Schematic showing the experimental setup used in this study. Reagents are delivered using syringe pumps and a selector valve. Two pressure sensors, one for the control layer and one for the fluidic layer monitor pressure inside channels and enable control of flow-rate as required.

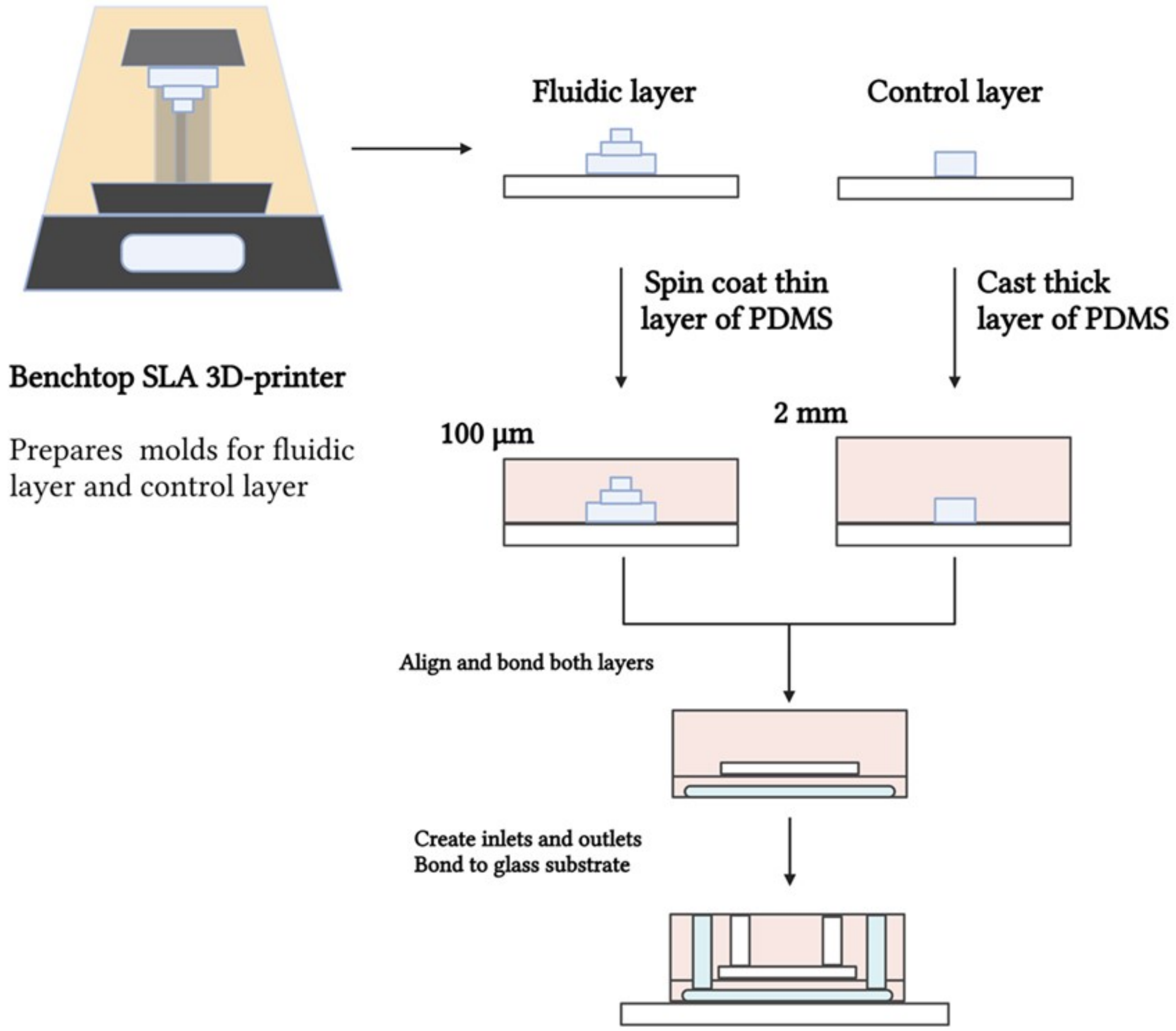

Figure S7: Schematic showing the benchtop preparation of ACBC devices from 3D printed moulds. Both fluidic and control layers are printed, cast with a thin and a thick layer of PDMS respectively and finally aligned and bonded so as to form the final device architecture.

Assay Characterisation

The assay identification results are shown as a confusion matrix (**fig. 4b**), which make use of the following terms:

- True positive (TP): Single bacterial cells identified as the correct species identity.
- False positive (FP): Single bacterial cells incorrectly identified as a different species identity.
- True negative (TN): Single bacterial cells correctly identified as negative to a particular species identity.
- False negative (FN): Single bacterial cells incorrectly identified as negative.

Sensitivity towards a particular species was calculated as the ability of the assay to correctly identify positive cells of that particular species. It was calculated by dividing the number of true positives over the total number of positives within that species identity:

$$Sensitivity = \frac{TP}{TP + FN}$$

Specificity refers to the ability of the assay to correctly identify negative single bacterial cells to a particular species identity. It was calculated by dividing the number of true negatives over the total number of positives of a particular species identity:

$$Specificity = \frac{TN}{TN + FP}$$

The percentages of single bacterial cells that are correctly and incorrectly predicted by the assay are given by the positive predictive value (PPV) and negative predictive value (NPV), respectively:

$$PPV = \frac{TP}{TP + FP}$$

$$NPV = \frac{TN}{TN + FN}$$

Minimum number of single bacterial cells required to make a positive call

To calculate the number of single-bacterial cells needed to inform as to the presence of a given pathogen (from the panel of 7 pathogens) we calculated the probability of this scenario such that:

$$P(N \geq 1) = 1 - P(N = 0) = 1 - (1 - PPV)^n$$

Where *n* is the total number of positive calls for a particular species observed, *N* is the number of true positives within those calls, $P(N \geq 1)$ is the probability that one or more true-positive cells are present, $P(N = 0)$ is the probability that no true-positive species are present. To estimate the presence of the particular species with 99.5% confidence, the following condition was solved for *n*:

$$1 - (1 - PPV)^n \geq \alpha$$

Where α is the desired level of confidence (in this case α = 0.995).

The probability of having exactly *k* positive identifications in *n* number of cells isolated and assayed is given by:

$$P(N = k) = \binom{n}{k} TP^k (1 - TP)^{n - k}$$

Where TP is the true-positive rate of identification for a particular species. To calculate the number of bacterial cells needed to be isolated by the ACBC chip to obtain a sufficiently high probability to observe at least the minimum number of positive calls required for identification (assuming a single-pathogen infection) was finally calculated using the following:

$$P(N \geq n_{positive}) = 1 - \sum_{k = 0}^{n_{positive} - 1} \binom{n}{k} TP^k (1 - TP)^{n - k}$$

The above equation was solved for the value of *n* that yielded a probability greater than 99.5% to estimate the number of cells of a given species that would be required to be isolated ($n_{isolated}$) and assayed by the device in order to give a positive call as to the presence of the particular pathogen in the sample. Tabulated results of the assay characteristics and minimum number of required cells can be found in **Table S4**.

Table S1: Table containing the encoded FISH probes used for species identification.

ID	Species	Sequence (5′→3′)	Ref
1	*Escherichia coli*	ACCGGTGACGTTAGATTAAAGCTGGGCAAAGGTATTAACTTTACT CCCTTCCTCCCGGGATGCCCCGATTATTGCGTCAT	1
2	*Klebsiella pneumoniae*	ACCGGTGACGTTAGATTAAAGCTGGAGAGCAAGCTCTCTGTGCTA CCGCTCGACTGGTTACATGACTGTCCGTGCTACTC	2
3	*Pseudomonas aeruginosa*	GCCCCTACAGTTCGCAAGTCAGTTAGTTTCCGGACGTTATCCCCCA CTACCAGGCAGTACACCCACCTAGGTCTTGGATG	1
4	*Enterococcus faecalis*	ACCGGTGACGTTAGATTAAAGCTGGCAAGTGTTATCCCCCTCTGAT GGGTAGGTTAGTACACCCACCTAGGTCTTGGATG	1
5	*Streptococcus pneumoniae*	GCCCCTACAGTTCGCAAGTCAGTTACTGGTAGTGATGCAAGTGCA CCTTTTAAGCAGTCTGGTGGACGCAACATTTATAC	2
6	*Streptococcus agalactiae*	GCCCCTACAGTTCGCAAGTCAGTTATCTAGTGTAAACACCAAACCT CAGCGTTCTACGGTTACATGACTGTCCGTGCTACTC	2
7	*Staphylococcus aureus*	ACCGGTGACGTTAGATTAAAGCTGGCATCAGAGAAGCAAGCTTCT CGTCCGTTCGAGTCTGGTGGACGCAACATTTATAC	1

Table S2: Table containing the imager FISH probes used for species identification.

ID	Probe name	Sequence (5′→3′)
1	I1	**Cy5-**CCAGCTTTAATCTAACGTCACCGGT
2	I2	**Cy5-**TAACTGACTTGCGAACTGTAGGGGC
3	I3	**Cy5-**GTATAAATGTTGCGTCCACCAGACT
4	I4	**Cy5-**CATCCAAGACCTAGGTGGGTGTACT
5	I5	**Cy5-**ATGACGCAATAATCGGGGCATCCCG
6	I6	**Cy5-**GAGTAGCACGGACAGTCATGTAACC
7	EUB	**Cy5-**GCTGCCTCCCGTAGGAGT

Table S3: Tabulated species identification rates – Individual strain breakdown.

	ECOL	KLPN	PSAR	EFAEC	SPN	SAGA	SAUR	Inconclusive
ECOL1	0.99	0	0	0	0	0	0	0.01
ECOL2	0.99	0	0	0	0	0	0	0.01
ECOL3	0.99	0	0	0	0	0	0	0.01
ECOL4	0.92	0	0	0	0	0	0	0.08
KLPN1	0	0.99	0	0	0	0	0	0
KLPN2	0	0.99	0	0	0	0	0	0.01
KLPN3	0	0.94	0	0	0	0	0	0.05
KLPN4	0	0.99	0	0	0	0	0	0
PSAR1	0	0	0.93	0.01	0.02	0	0	0.04
PSAR2	0	0	0.77	0.05	0.05	0	0	0.12
PSAR3	0	0	0.91	0.02	0.02	0	0	0.05
PSAR4	0	0	0.87	0	0	0	0	0.12
EFAEC1	0	0	0.01	0.94	0	0	0	0.04
EFAEC2	0.01	0.01	0	0.95	0	0	0.01	0.03
EFAEC3	0	0	0	0.97	0	0	0	0.03
EFAEC4	0	0	0	0.99	0	0	0	0.01
SPN1	0	0	0	0	0.87	0.01	0.01	0.11
SPN2	0	0	0	0	0.65	0	0.13	0.22
SPN3	0	0	0	0	0.54	0	0.22	0.24
SPN4	0	0	0	0	0.73	0.01	0.07	0.19
SAGA1	0	0.04	0.02	0	0.02	0.73	0	0.19
SAGA2	0.01	0.08	0	0	0	0.72	0	0.19
SAGA3	0	0.1	0	0	0	0.86	0	0.04
SAGA4	0	0.01	0	0	0	0.93	0	0.06
SAUR1	0	0	0	0	0.06	0	0.87	0.07
SAUR2	0	0	0	0	0.1	0.01	0.76	0.12
SAUR3	0	0	0	0	0.02	0	0.94	0.04
SAUR4	0	0	0	0	0.01	0	0.98	0.01

Table S4: Identification assay characteristics per species including number of positive single-cell calls that would need to be made per species to reach 99.5% confidence ($n_{positive}$) of the species presence in the sample as well as the number of cells that would need to be isolated such that a sufficient number of cells have been collected to then perform a positive call 99.5% of the time ($n_{isolated}$).

Species	Sensitivity	Specificity	TP	PPV	$n_{positive}$	$n_{isolated}$
E. coli	0.98	1	0.98	1	1	2
K. pneumoniae	0.98	1	0.98	1	1	2
P. aeruginosa	0.91	0.99	0.87	0.96	2	5
E. faecalis	0.97	1	0.96	0.99	2	3
S. pneumoniae	0.79	0.98	0.70	0.86	3	9
S. agalactiae	0.87	0.99	0.81	0.92	3	7
S. aureus	0.94	0.99	0.89	0.95	2	4

Table S5: Performance characteristics of state-of-the-art rapid bacterial identification platforms employing a variety of detection modules. Performance is colour-coded in terms of desirability with dark-green as 'best performance', light-green as 'very good' and red as 'moderate'. Based on the below characteristics, the ACBC device excels in several areas, particularly in terms of limit-of-detection (LOD), time to identification, number of species in the assay panel, as well as its ability to perform ASTs in tandem with identification.

Platform	Detection	Enrichment Technology	Sample	No. Species	Multiplex	LOD (CFU/mL)	Time (mins)	Sample volume	AST	Device simplicity
ACBC	Microscopy and 16S FISH	Hydrodynamic Trapping by Adaptive Channels	PBS spike	7	Yes	7×10^2	60	42	Yes	Benchtop Fabrication
Kandavalli et al.[3]	Microscopy and 16S FISH	Hydrodynamic Trapping by Nanostructured Filters	Culture medium	7	Yes	1×10^4	105	--	Yes	Ion Beam Lithography
Shen et al.[4]	MALDI TOF MS	Herringbone structures and off-chip MALDI	PBS spike	5	Yes	1×10^4	90	250	No	Soft lithography
Fang et al.[5]	PCR	FcMBL-coated beads with PDMS membrane and PCR module	Blood spike	5	Yes	5	240	5400	No	Benchtop Fabrication
Berger et al.[6]	LAMP	CLIP-based AM PTFE capillary with integrated LAMP	Blood spike	1	No	50	45	8	No	Soft lithography
Rodriquez-Lorenzo et al.[7]	Raman	Hydrodynamic Focusing with tagged SERS enhancers	Buffer spike	2	No	1×10^5	30	50	No	Soft lithography

Table S6: Table containing the cost-breakdown for the components needed to setup the fluidic system used.

Item	*Number*	*Unit Cost (GBP)*	*Cost (GBP)*
Syringe pump components			
NEMA17 motor	9	10.12	91.08
M5 x 90 mm Thread	9	0.865	7.78
syringe body 3d printed	9	1.36	12.24
hex nuts and threads			1.4
Arduino Uno	2	21.2	42.4
Arduino CNC Shield	2	12.99	25.98
Labsmith			
Sensor Manifold	2	150.3538	300.7076
Pressure Sensors	2	159.7622	319.5244
Tee Interconnect. Ultem® 1/16"	2	22.5492	45.0984
1x valve starter package kit	1	1718.5466	1718.5466
1x 8-port valve	1	1012.4092	1012.4092
3D Printing			
Anycubic Mono 4k	1	155.83	155.83
Anycubic Plant-based ECO resin (clear)	1	18	18
		**Total**	**3759.66**

References

1 G. J. Jansen, M. Mooibroek, J. Idema, H. J. M. Harmsen, G. W. Welling and J. E. Degener, *Rapid Identification of Bacteria in Blood Cultures by Using Fluorescently Labeled Oligonucleotide Probes*, 2000, vol. 38.

2 K. V. A. J, T. Karlheinz and A. I. B, *J Clin Microbiol*, 2000, **38**, 830–838.

3 V. Kandavalli, P. Karempudi, J. Larsson and J. Elf, *Nat Commun*, , DOI:10.1038/s41467-022-33659-1.

4 Y. Shen, J. Yi, M. Song, D. Li, Y. Wu, Y. J. Liu, M. Yang and L. Qiao, *Analyst*, 2021, **146**, 4146–4153.

5 Y. L. Fang, C. H. Wang, Y. S. Chen, C. C. Chien, F. C. Kuo, H. L. You, M. S. Lee and G. Bin Lee, *Lab Chip*, 2021, **21**, 113–121.

6 J. Berger, M. Y. Aydin, R. Stavins, J. Heredia, A. Mostafa, A. Ganguli, E. Valera, R. Bashir and W. P. King, *Anal Chem*, 2021, **93**, 10048–10055.

7 L. Rodríguez-Lorenzo, A. Garrido-Maestu, A. K. Bhunia, B. Espiña, M. Prado, L. Diéguez and S. Abalde-Cela, *ACS Appl Nano Mater*, 2019, **2**, 6081–6086.
